# A Comparison of Cognitive Function in Pine Study to Health and Retirement Study & CHARLS Study

**DOI:** 10.1093/geroni/igaa057.3390

**Published:** 2020-12-16

**Authors:** Charu Verma, Mailun Zhang, Mengting Li, Stephanie Bergren, XinQi Dong

**Affiliations:** Rutgers University, New Brunswick, New Jersey, United States

## Abstract

Healthy immigrant theory assumes that immigrants have better health than natives, while it remains unclear whether this theory applies to older Chinese Americans with respect to cognitive health. The objective of this study was to estimate the differences in cognitive function between the US and Chinese older adults. PINE (n=3,157) (2011-2013) with Chinese older adults was compared to HRS study (2010-2011) (n=22,034) and HRS sister CHARLS (n=17,708) (2010-2011). Cognitive function was assessed by episodic memory, working memory, executive function, and MMSE. Cognition impairment was defined by 1.5 standard deviations below mean of z scores. After 1:1 matched samples determined by propensity score and verified by McNemar and Cohen’s Kappa test, conditional logistic regression model with demographic variables controlled was used to assess the differences between the three studies. Multivariable analyses results showed that participants from PINE are 5.667 (OR= 5.667, 95% CI 3.893-8.248, p < .001) times more likely to have cognitive impairment in comparison to HRS’s and 0.166 (OR= 0.166, 95% CI 0.136-0.203, p < .001) times less likely in comparison to CHARLS’s. Result demonstrated that Chinese older adults living in the US have a higher likelihood of cognitive impairment in comparison to the US older adults but less likely than those living in China. Healthy immigrant theory is partially supported in Chinese Americans. Immigrants with better cognitive health are likely to migrate to the US, but limited social engagement in the receiving communities might be a risk factor for cognitive function, leading to worse cognitive health than natives.

